# A rare case of spontaneous hepatic rupture in a pregnant woman

**DOI:** 10.1186/s12884-018-1713-5

**Published:** 2018-04-10

**Authors:** Xiao Zhou, Meng Zhang, Zhuang Liu, Meili Duan, Lei Dong

**Affiliations:** Department of Intensive Care Unit, Beijing Friendship Hospital, Capital Medical University, No. 95 Yong-An Road, Xi Cheng District, Beijing, 100050 China

**Keywords:** Perinatal mortality, Pregnancy complications, Rupture, Spontaneous, Fatty liver

## Abstract

**Background:**

Spontaneous hepatic rupture in pregnancy is a rare and life-threatening event during the perinatal period.

**Case presentation:**

We report a case of a 33-year-old woman with 36 + 6 weeks’ gestation that present with elevated blood pressure before delivery, who was admitted to our hospital due to irregular abdominal pain. Diagnosed with abdominal paracentesis, the emergent caesarean section and laparotomy were performed. Postoperatively, the patient experienced 22-day intensive therapy in ICU and was transferred to the General Surgery Department in good physical condition without post-operative complications.

**Conclusions:**

This case indicates that making an accurate and timely diagnosis and taking multidisciplinary approach contribute to a successful clinical outcome.

## Background

Spontaneous hepatic rupture in pregnancy is a rare condition and carries a high maternal and perinatal mortality [1]. It is usually associated with hepatic hemangiomata, hepatic metastases from choriocarcinoma, or some unknown causes. Its sudden appearance and potentially fatal outcome make it an important diagnostic and therapeutic challenge.

We reported a successfully treated case of spontaneous hepatic rupture in a pregnant woman. It emphasizes the need for the early diagnosis as well as the timely surgical management and the postoperative monitoring of patients in the intensive care unit. The relevant literature is also reviewed.

## Case presentation

A 33-year-old primigravida woman, a body mass index of 31 kg/cm2, was admitted to the emergency department at36 + 6 weeks gestation with abdominal pain that had lasted for 6 h. She did not have a history of trauma. Her antenatal period had been uneventful except for one episode of hypertension (141/84 mmHg). Physical examination revealed a blood pressure of 102/62 mm/Hg, a heart rate of 80 beats/min and a temperature of 36.6 °C. Blood tests showed hemoglobin of 95 g/L,Hematocrit of 28.9%, platelet count of 111 × 10^9^/L(reference range, 110-300 × 10^9^/L), reticulocyte count of 0.0525 × 10^12^/L(reference range, 0.014-0.09 × 10^12^/L), liver enzymes with an alanine aminotransferase of 281 U/L, aspartate aminotransferase of 392 U/L, lactate dehydrogenase of 525 U/L, total bilirubin levels of 19 μmol/L(reference range, 3.42-17.1umol/L), and normal alpha fetoprotein. Serological markers of hepatitis B and hepatitis C virus were negative. Ultrasound scan showed that fetal heart beats could not be found and free abdominal fluid was present. There were no images suggesting placental abruption. Ultrasound-guided intra-abdominal puncture revealed active intraperitoneal bleeding.

In less than 3 h of admission, an emergency laparotomy was performed and a dead fetus was delivered by cesarean section. No evidence of placental abruption or active intrauterine bleeding was detected. Exploration of the ovarian tubes and ovaries revealed no bleeding or neoplasm. Two liters of hemoperitoneum was removed from abdominal cavity. Active hemorrhage came from rupture of the right lobe of the liver which appeared fatty liver. Direct hemostasis was not achievable because the liver was edematous and was ruptured during the performance of sutures. Thus perihepatic packing was done by seven large gauze packs and hemostatic sponges were used to compress the bleeding lesions. Throughout the laparotomy, intraoperative bleeding was 2000 mL and the patient received 8 units of RBC and 1200 ml of plasma.

The patient was transferred to the ICU after surgery to continue resuscitation with intravenous fluids and vasoactive support. The following day in ICU, she had high fever and developed the acute kidney injury and severe acute respiratory distress syndrome. She was managed by massive blood transfusion, mechanical ventilation, renal replacement therapy and antibiotic treatment. Intra-abdominal pressure was maintained between 7.5 and 11.3 mmHg (1–1.5 KPa) by abdominal binder.

**Fig. 1:**
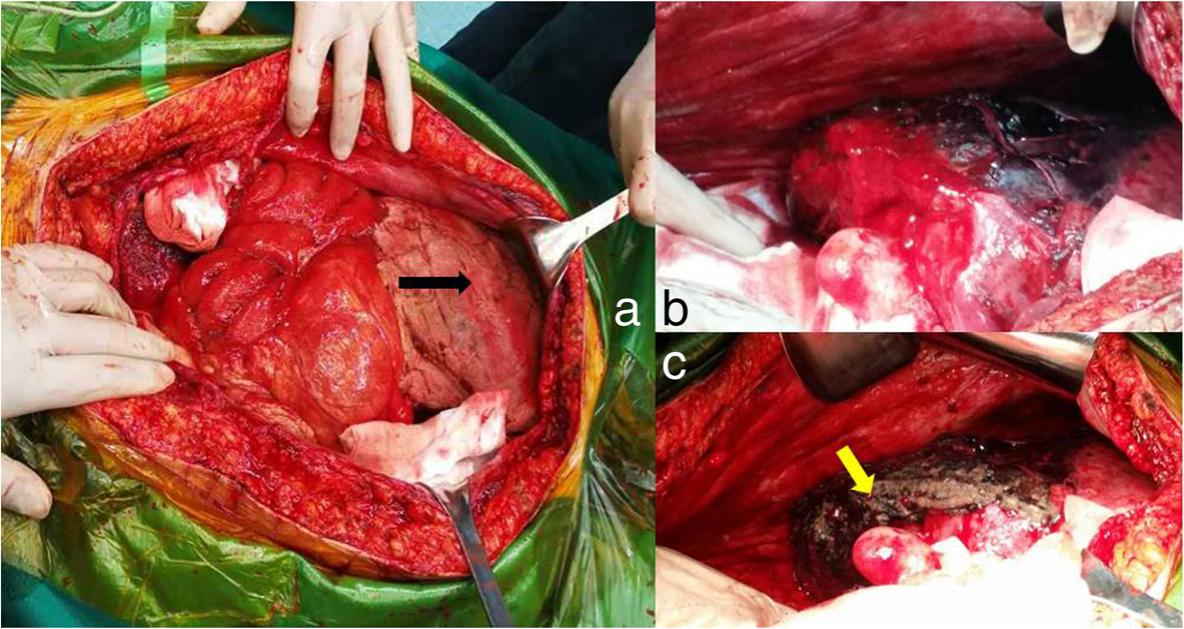
The 2nd operation findings. **a** liver packing (black arrow shows gauzes). **b** liver hemotoma. **c** Argon laser to stop bleeding (yellow arrow shows laser-coagulation)

**Fig. 2:**
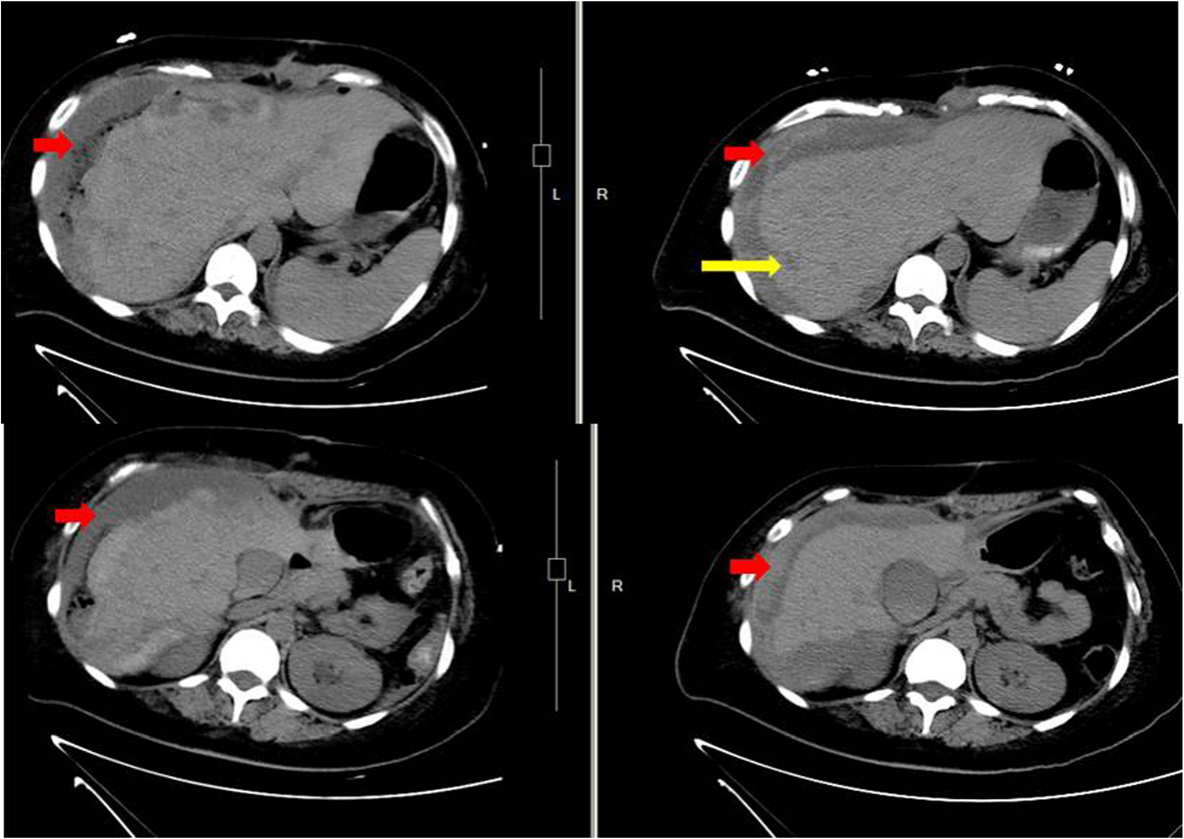
Abdominal CT scans on the 3rd day (left) and 13th day (right) post the second surgery. Red arrows (hypodense collection) show perihepatic effusion. Yellow arrow shows the ruptures of the liver

A second laparotomy was performed on the 6th postoperative day. A large hematoma and rupture of the right liver lobe were visible after removing the gauze packs (Fig. 1a), which was still bleeding (Fig. 1b). The patient was treated with argon laser to stop bleeding (Fig. 1c). Moreover, one drain was left in the abdomen. Postoperatively, ultrasound showed perihepatic and pelvic free fluid that required percutaneous drainage. Abdominal computed tomography (CT) scan (Fig. 2) revealed perihepatic effusion with laceration of the right liver lobe and hypo-density of fluid in the peritoneal cavity. Decreasing in volume of the perihepatic effusion was observed by subsequent CT scan (Fig. 2). After 22 days of recovery in ICU, the patient was transferred to the surgical department where she remained for further 3 weeks with regular post-operative course. She was discharged in normal physical condition after 45 days.

## Discussion and conclusions

Spontaneous hepatic rupture in pregnancy, which was first described by Abercrombie in 1984, is a rare condition associated with high maternal and perinatal mortality [1], with its incidence ranging from 1 in 45,000 to1 in 225,000 deliveries [2]. A recent literature review of hepatic rupture revealed a maternal mortality rate of 39% [3] and a perinatal mortality rate of 42% [4]. Spontaneous hepatic rupture is commonly caused by hepatic hemangiomata, hepatocellular carcinoma, hepatic adenoma, of which were reported to be pregnancy-related but rare [5]. Other causes arehepatic metastases from choriocarcinoma, focal nodular hyperplasia, peliosishepatis, trauma, infection (malaria, syphilis, amoebic abscess), aneurysms; granulomas, the use of cocaine during pregnancy, anabolic steroid therapy, systemic amyloidosis [6] and unknowns [7, 8]. Hepatic rupture in pregnancy usually is associated with pre-eclampsia, eclampsia, or Hemolysis Elevated Liver enzymes and Low Platelet count (HELLP) syndrome [9]. Spontaneous hepatic rupture in uncomplicated pregnancy also has been reported but is extremely rare [10]. The presenting symptoms and signs of liver bleeding are subtle and non-specific, the most common are abdominal pain, shoulder pain and vomiting. Thus, a high index of clinical suspicion is necessary and emergency ultrasonography, CT, or magnetic resonance imaging should be considered to reach the diagnosis of spontaneous hepatic rupture in a timely manner. As a pregnant woman presents with a sudden onset of unexplained, severe abdominal pain, spontaneous rupture of the liver should be suspected.

Hepatic rupture requires immediate laparotomy to stop bleeding, therapeutic options involves perihepatic packing, placement of sponges or absorbable mesh, fibrin glue, argon laser coagulation and hepatic artery ligation. Partial liver resection or even liver transplantation has been described in the literatures. The outcome of patients with spontaneous hepatic rupturegreatly depends on the etiology and severity of the hemorrhage [11, 12].

In this case, the patient had no history of trauma, excessive alcohol consumption, or use of hepatotoxic medications, moreover, she did not have pre-eclampsia or eclampsia during this pregnancy, tumor biomarkers as well as clinical tests of hepatitis B and C virus infection were negative. Liver lesions were not visible at operation or with imaging. There was insufficient evidence for the diagnosis of HELLP syndrome: the platelet count was marginally low even with massive hemorrhage, tests of hemolysis were negative (serum indirect bilirubin, reticulocyte count, Coombs test, and Ham’s test), and liver enzymes before acute hemorrhage were mildly elevated. Nevertheless, HELLP syndrome cannot be totally excluded due to the presence of hypertension. HELLP syndrome is considered as a risk factor for hepatic rupture in pregnancy. Vigil-De et al. reported that it was present in 92.8% of cases in their series during the past two decades [12]. With this case of a high BMI, fatty liver was suspected during laparotomy but no evidence from the biopsy yet. A few cases of fatty liver disease associated with hepatic rupture have been reported, the incidence was about 1 in 13,000 pregnancies [13].

A large hematoma was on the right lobe of the liver in this patient. Perihepatic packing was carried out to control the bleeding in this case, however, she remained at risk for complications, such as intraperitoneal infection. For patient with continued bleeding after the first surgical operation, ICU assistance played a valuable role on the recovery during the post-operative course. The right hepatic lobectomy was another choice for the surgeon when hemorrhage remained uncontrolled, which probably led to postoperative liver dysfunction. Hence, an effort should be made to gain hemostatic control while avoiding large resections as much as possible.

In conclusion, spontaneous hepatic rupture in pregnancy is rare but life-threatening. A high level of alertness for the diagnosis aided by timely ultrasonography and CT scan is critical to prompt intervention, which requires multidisciplinary approach to achieve a successful outcome.

## Abbreviations

CT: Computer tomography
HELLP: Syndromehemolysis elevated liver enzymes and low platelet syndrome
ICU: Intensive care unit
